# The effects of stimulation of the autonomic nervous system via perioperative nutrition on postoperative ileus and anastomotic leakage following colorectal surgery (SANICS II trial): a study protocol for a double-blind randomized controlled trial

**DOI:** 10.1186/s13063-014-0532-x

**Published:** 2015-01-27

**Authors:** Emmeline G Peters, Boudewijn JJ Smeets, Marloes Dekkers, Marc D Buise, Wouter J de Jonge, Gerrit D Slooter, Tammo S de Vries Reilingh, Johannes A Wegdam, Grard AP Nieuwenhuijzen, Harm JT Rutten, Ignace HJT de Hingh, Mickael Hiligsmann, Wim A Buurman, Misha DP Luyer

**Affiliations:** Department of Surgery, Catharina Hospital, Michelangelolaan 2, 5623 EJ Eindhoven, The Netherlands; Academic Medical Center, Tytgat Institute for Intestinal and Liver Research, Department of Gastroenterology, Meibergdreef 69-71, 1105 BK Amsterdam, The Netherlands; Department of Anesthesiology, Catharina Hospital, Michelangelolaan 2, 5623 EJ Eindhoven, The Netherlands; Department of Surgery, Maxima Medical Center, De Run 4600, 5504 DB Veldhoven, The Netherlands; Department of Surgery, Elkerliek Hospital, Wesselmanlaan 25, 5707 HA Helmond, The Netherlands; Department of Health Services Research, Maastricht University, Duboisdomein 30, 6229 GT Maastricht, The Netherlands; Institute MHeNS, Maastricht University, PO Box 616, 6200 MD Maastricht, The Netherlands

**Keywords:** Perioperative nutrition, Colorectal surgery, Postoperative ileus, Anastomotic leakage, Autonomic nervous system, Inflammation

## Abstract

**Background:**

Postoperative ileus and anastomotic leakage are important complications following colorectal surgery associated with short-term morbidity and mortality. Previous experimental and preclinical studies have shown that a short intervention with enriched enteral nutrition dampens inflammation via stimulation of the autonomic nervous system and thereby reduces postoperative ileus. Furthermore, early administration of enteral nutrition reduced anastomotic leakage. This study will investigate the effect of nutritional stimulation of the autonomic nervous system just before, during and early after colorectal surgery on inflammation, postoperative ileus and anastomotic leakage.

**Methods/Design:**

This multicenter, prospective, double-blind, randomized controlled trial will include 280 patients undergoing colorectal surgery. All patients will receive a selfmigrating nasojejunal tube that will be connected to a specially designed blinded tubing system. Patients will be allocated either to the intervention group, receiving perioperative nutrition, or to the control group, receiving no nutrition. The primary endpoint is postoperative ileus. Secondary endpoints include anastomotic leakage, local and systemic inflammation, (aspiration) pneumonia, surgical complications classified according to Clavien-Dindo, quality of life, gut barrier integrity and time until functional recovery. Furthermore, a cost-effectiveness analysis will be performed.

**Discussion:**

Activation of the autonomic nervous system via perioperative enteral feeding is expected to dampen the local and systemic inflammatory response. Consequently, postoperative ileus will be reduced as well as anastomotic leakage. The present study is the first to investigate the effects of enriched nutrition given shortly before, during and after surgery in a clinical setting.

**Trial registration:**

ClinicalTrials.gov: NCT02175979 - date of registration: 25 June 2014.

Dutch Trial Registry: NTR4670 - date of registration: 1 August 2014.

## Background

Postoperative ileus (POI) is considered to be an inevitable consequence of colorectal surgery by many clinicians and is associated with an increased morbidity and prolonged hospital stay. Therapeutic strategies to cure this condition of decreased gastrointestinal motility in our current health system are lacking and at this moment only supportive care is given to optimize the patient’s condition. Although perioperative care is improved by several measures, such as encouraging early mobilization, minimally invasive surgery and standard anesthetic protocols as part of the Enhanced Recovery After Surgery (ERAS) guidelines, POI cannot be completely avoided and the incidence remains substantial [1,2]. POI is caused by a local and systemic inflammatory response associated with bowel manipulation [3,4]. In experimental studies, it is shown that nutritional stimulation of the autonomic nervous system reduces the inflammatory response and POI via a specific mechanism [5-7]. Ingestion of dietary lipids, proteins and peptides triggers release of cholecystokinin (CCK) via chylomicron formation [8]. CCK binds to CCK-1 receptors located on vagal afferents, thereby rapidly activating the autonomic nervous system and subsequently activating efferent vagal nerve fibers leading to the release of acetylcholine. Acetylcholine then binds to alpha-7-nicotinic receptors, located on inflammatory cells such as mast cells, triggering an intracellular pathway that results in a decreased release of inflammatory mediators [9-11]. Via this mechanism, POI can be inhibited [9,10].

Also, in previous preclinical and clinical studies, our group has shown that enteral nutrition and sham-feeding via chewing gum are very effective means in the reduction of POI and inflammation. When enteral nutrition was started very early after colorectal surgery, POI was reduced from 35% to 16%. In a second study, using chewing gum as a means to stimulate the autonomic nervous system, a reduction of POI was seen from 48% in the control group to 27% in the intervention group [12,13]. These studies also revealed an as yet unaccountable effect on anastomotic leakage (AL), resulting in an average reduction of 73%. AL is a dreaded complication following colorectal surgery, which is associated with significant morbidity and even mortality. It is a risk factor for local recurrence of colorectal cancer and has a significant impact on overall survival and quality of life [14].

Composition of the enteral nutrition and timing of administration are essential for the magnitude of this response. Administration of an enriched enteral formula that optimally stimulates release of CCK via chylomicrons just before, during and directly after the inciting event for inflammation is pivotal [9]. The current study will investigate whether enriched enteral nutrition given shortly before, during and early after colorectal surgery reduces POI and AL via stimulation of the autonomic nervous system in a clinical setting. Given limited healthcare resources and the increasing importance of economic evaluation, we also plan to estimate the cost-effectiveness of this intervention in the current medical healthcare system.

### Study objectives

The objective is to investigate whether enriched enteral nutrition given shortly before, during and early after colorectal surgery, reduces POI and AL via stimulation of the autonomic nervous system in a clinical setting, compared with standard care. The primary outcome will be POI. Secondary outcomes will include effects of perioperative nutrition compared with standard of care on AL, aspiration pneumonia, gastric volumes preoperatively, time until functional recovery, inflammatory markers, intestinal barrier integrity, surgical complications according to Clavien-Dindo and health-related quality of life. Furthermore, a cost-effectiveness analysis and a cost-utility analysis will be performed.

## Methods/Design

This is a multicenter, prospective, double-blind, randomized controlled superiority trial with two parallel groups. A written informed consent needs to be obtained from every patient. The trial will be conducted according to the rules of Good Clinical Practice, and a Data Safety Monitoring Board (DSMB) is installed to monitor (serious) adverse events. Ethical approval for this study was granted by the Medical Ethics Committee Catharina Hospital (Eindhoven, The Netherlands) under reference number NL45640.060.13.

### Study population

**Figure 1:**
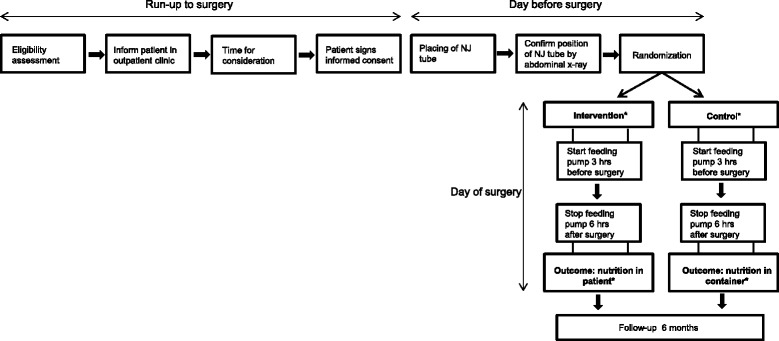
**Flow diagram for participants who will be included in the study.** *Blinded groups and blinded outcome. NJ tube = nasojejunal tube.

Patients aged >18 undergoing elective segmental colorectal resection with an anastomosis are eligible for inclusion. A total of 280 patients will be included. Exclusion criteria are previous gastric or esophageal resection, peritoneal carcinomatosis, pre-existent or creation of an ileostomy, steroid use and use of medication that disrupts the acetylcholine metabolism, such as selective serotonin reuptake inhibitors and anticonvulsants. Both the investigator and the responsible surgeon will verify eligibility. If the indication for operation is established, the patient will receive written and oral information about the trial during a scheduled outpatient appointment. Patients will be offered sufficient time to enquire about details of the trial and to decide whether or not they wish to participate. Patients will have to sign the informed consent form in the physical presence of the surgeon or investigator. A participant flow diagram is shown in Figure 1.

### Participating centers

Patients eligible for this study will be initially included in the Catharina Hospital, Eindhoven, The Netherlands. After inclusion of 40 patients, a safety analysis will be performed to assess feasibility and safety of perioperative nutrition. After this first safety analysis, this study will be conducted in a multicenter setting. Also, when 140 patients are included, an interim-analysis will be performed. The estimated duration of the recruitment period is 2 years.

### Randomization

Patients will be randomized as block randomization with a 1:1 allocation by means of randomization software (TENALEA Clinical Trial Data Management System, Amsterdam, The Netherlands). A selfmigrating nasojejunal tube (Bengmark, Nutricia Flocare, Zoetermeer, The Netherlands) will be inserted in all included patients. To minimize aspiration, the nasojejunal tube needs to be positioned postpylorically, which will be confirmed by an abdominal X-ray. The patient will be randomized after confirmation of a correct position of the nasojejunal tube. Blinded stratification will be applied to ensure an equal distribution between colonic or rectal surgery and between a laparoscopic or an open procedure.

After randomization, enriched enteral nutrition (Nutricia Research, Utrecht, The Netherlands) will be started via an electronic feeding pump (Infinity, Nutricia Flocare, Zoetermeer, The Netherlands) in all patients. The nutrition and the pump will be connected to a specially designed opaque tubing system (ECMbv, Gemert, The Netherlands) that is similar to what is used in daily practice. However, the tubing has a bifurcation with two branches; one branch leads to the nasojejunal feeding tube in the patient and one branch leads to a sealed container (attached to the bed in all patients). For patients who will be allocated to the intervention group, the tubing system is open towards the patient and occluded towards the container. For patients in the control group, the tubing system is occluded towards the patient and open towards the container. In this way, the patients in the intervention group will receive enriched enteral nutrition, while the patients in the control group will not. The tubing will be labeled with a randomization number provided by TENALEA. Independent research nurses will provide the labeling of the tubing and store the allocation list.

The tubing system can easily be connected to all materials, including the standard enteral feeding system, the nasojejunal feeding tube, and the container. The containers will be sealed and weighted to prevent differentiation between the groups by examining the containers. Neither the patient, nor the nursing staff or the researchers will be able to see which branch of the tubing system is occluded.

### Study outline

The day before surgery, all patients will receive a selfmigrating nasojejunal feeding tube at the ward. The position of the nasojejunal tube will be verified by means of abdominal X-ray. Preoperatively, all patients will be fasted for oral solid foods for a period of 6 hours, and oral liquids for a period of 2 hours. Three hours before surgery, the pump with enriched tube feeding will be started at a continuous rate of 1.5 ml/minute in all patients. Subsequently, 6 hours after surgery, the feeding pump will be stopped and the nasojejunal tube will be removed. Depending on the randomization outcome, the nutrition will be administered either to the patient, or into the sealed container. This setup allows a double-blind comparison between the control group and the intervention group, receiving enriched enteral nutrition. The fixed timeframe and the administration of feeding by electronic pump will support adherence to the study protocol. Anesthetic regimens will be standardized and registered per patient, as will the standardized postoperative care. Participants should continue to take medication for other conditions as usual.

Both the rate of infusion and the composition of the nutrition have been described in previous studies [9]. Furthermore, the nutrition has been tested in healthy volunteers and is well tolerated [9]. The enriched nutrition contains 44 energy percent (en%) fat, 25 en% protein, and 31 en% carbohydrates. The protein fraction consists of intact casein, whey protein, and soy protein hydrolysate. The lipid fraction contains less than 5 weight percent omega-3 fatty acids.

Perioperative nutrition is a new concept with a potential risk of aspiration pneumonia. In this trial, nutrition will be administered postpylorically, thereby reducing gastric volumes. To minimize risk of aspiration pneumonia the stomach will be emptied by means of a gastric tube before surgery. Gastric volumes will be measured in every patient when the stomach is emptied before surgery in the operating room. By giving the nutrition postpylorically, in a low volume and assuring an empty stomach before intubation, the risk of complications will be minimized.

A Data Safety Monitoring Board (DSMB) has been installed to evaluate all complications and monitor patient safety. If a serious event regarding safety of preoperative feeding (for example, aspiration pneumonia) occurs, the DSMB will be informed, who will then discuss the consequences and report their advice to the investigator and Medical Ethics Committee.

POI is defined in accordance with a standardized definition, and will be measured clinically. POI is established if both of the following criteria are met: lack of passage of flatus or stool, and inability to tolerate an oral diet in the interval between surgery and postoperative day 4 [15].

Prolonged POI is established if 2 or more of the following criteria are met on or after postoperative day 4 without prior resolution of POI: nausea or vomiting, inability to tolerate an oral diet over the last 24 hours, absence of flatus over the last 24 hours, abdominal distension, and/or radiologic confirmation.

In addition, gastric motility will be measured by means of ultrasound of the gastric antrum before and after a standardized meal on the second postoperative day. Previous studies have shown that ultrasonic gastric antrum measurements are representative for determining gastric emptying and gastric motility by determining the change in the gastric antral cross sectional area of the stomach before a standardized meal, and 15 and 90 minutes postprandially [16].

AL is defined as clinical and radiological signs of anastomotic leakage, if confirmed by re-operation or if an enterocutaneous fistula occurs. Blood plasma, peritoneal lavage fluid and tissue samples will be collected at predefined time-points before, during and after surgery. All samples will be stored at −80°C until further analysis. These samples will be analyzed for markers of the inflammatory response and gut barrier integrity using immunohistochemistry, Western blot, ELISA and/or PCR.

Patient characteristics, nutritional status and clinical parameters will be registered prospectively. Surgical complications will be classified using the Clavien-Dindo classification [17]. Quality of life will be measured preoperatively and postoperatively at 3 and 6 months after surgery via checklists (EORTC QLQ C-30 in cancer patients and EuroQol-5D-5L (EQ-5D-5L)).

The total follow-up for this study is 6 months. All data will be registered on a paper case report form and in an electronic database. The principal investigator and the local investigators will have access to the dataset.

### Statistical analysis

The sample size is calculated with a power analysis and is aimed at POI and AL, based on previous studies of our group [12,13]. For POI, these previous data show a mean incidence of 40% and a reduction of almost 50% by nutrition or sham-feeding. Based on a power of 0.8 and an alpha of 0.05, a total of 91 patients are needed per group. For AL, our previous data show a mean incidence of 13% and a reduction to 3.5% by nutrition or sham-feeding. Using a power of 0.8 and a drop-out percentage of 5%, a total of 140 patients are needed per group. In this way both the primary endpoint and the most important secondary endpoint AL will be adequately powered and a multicomparison correction will not be applied in the final analysis.

All analyses will be done according to the intention-to-treat approach, which includes all randomized patients, regardless of adherence to study protocol. Occurrences of the primary and secondary endpoints will be compared between the intervention group and the control group. Results will be presented as risk ratios with corresponding 95% confidence intervals. A 2-tailed *P*-value < 0.05 is considered statistically significant. To compare the groups, the data will be tested for normal distribution, and an unpaired *t*-test will be performed if appropriate, otherwise the Mann-Whitney *U*-test, Chi-square test or Fisher's exact test will be used.

### Economic evaluation design

A cost-effectiveness analysis and a cost-utility analysis will be performed to assess the economic value of perioperative nutrition compared with usual care. The incremental cost-effectiveness ratio (ICER) will be expressed as the incremental costs per point change in POI and AL, and the incremental cost-utility ratio (ICUR) will be expressed as the incremental costs per quality-adjusted life years (QALYs) gained. Quality of life will be measured using the EORTC QLQ C-30 and EQ-5D-5L questionnaires.

A societal perspective will be used for cost evaluation. We will consider cost factors in three categories: healthcare sector costs (direct costs such as personnel, operation, or diagnostics), patient and family costs (out-of-pocket expenses, travel expenses), and costs in other sectors (mainly cost due to absenteeism in paid work and production losses in the domestic sphere). Healthcare costs, costs to patients and family, and costs occurring in other sectors will be measured at the individual (participant) level using data from registration systems of the hospitals and patient questionnaires (including adapted version of the iMTA Medical Consumption Questionnaire (iMCQ) and the iMTA Productivity Cost Questionnaire (iPCQ)). Finally, monetary values will be assigned to the relevant cost factors. The identified health services consumed by study participants will be multiplied with their corresponding unit prices. Total costs will be determined by summing up the costs of individual services. The updated Dutch manual for costing in economic evaluations in healthcare will be used in the valuation step of healthcare costs and patient cost. Costs will be indexed for the year 2014.

ICER (and ICUR) will be estimated as the difference in costs between perioperative nutrition and standard care divided by their differences in outcomes. To quantify the uncertainty around the ICER, non-parametric bootstrapping will be conducted in Excel (Microsoft, Redmond, WA, USA) (1,000 simulations). The results of the bootstrapping will be plotted in the cost-effectiveness plane, where the horizontal axis reflects differences in effects and the vertical axis differences in costs, and will be represented as a cost-effective acceptability curve showing the probability that perioperative nutrition is cost-effective for a range of willingness-to-pay values. Additional sensitivity analyses will be done to test the robustness of the results.

## Discussion

Despite advances that are being made in surgical technique and improvement in postoperative care, POI and AL remain very important clinical determinants of short-term morbidity and mortality following colorectal surgery. In previous studies it was shown that 35 to 45% and 7 to 13% of patients experience POI and AL after elective colorectal surgery, respectively [12,13]. Both POI and AL have proven to be significant predictors of increased hospital length of stay and hospital costs [2,18,19]. Furthermore, AL is a risk factor for local recurrence of colorectal cancer and has a significant impact on disease-free and overall survival [14]. It is, therefore, believed that improving postoperative outcome following colorectal surgery will also improve long-term oncological outcomes regarding overall survival and tumor recurrence [20].

Although the mechanism underlying POI has been studied extensively in animal experiments, targeted therapeutic strategies are currently lacking in a clinical setting and only supportive measures are taken to optimize the patient’s recovery [21]. For POI it is believed that formation of an inflammatory infiltrate in the muscular layers of the intestine following bowel manipulation during surgery leads to a decreased gastrointestinal motility [4]. In previous experimental and clinical studies our group has shown that a short intervention with enriched enteral nutrition given just before and directly after the inciting event, dampens the inflammatory response and reduces POI by stimulation of the autonomic nervous system through release of CCK. Evidence on the relation between POI and AL is scarce, but has a great impact. Early inhibition of the inflammatory response may play a role, since release of proinflammatory cytokines such as TNF-α modulates intestinal epithelial function and is an inhibitory factor in the wound healing process of intestinal anastomoses [22].

Nutrition is usually given to provide nutrients, or to administer specific nutrients with a potential therapeutic effect (for example, probiotics, omega-3 fatty acids) [23]. However, in this study enteral nutrition is used to directly alter the immune response following colorectal surgery via a newly discovered neuro-immunological feedback mechanism. This is a new concept and changes the way of thinking about the usage of enteral nutrition in a clinical setting.

To optimally stimulate the release of CCK and thereby activating the autonomic nervous system, a specific nutritional formula will be used that is enriched in lipids and proteins. Furthermore, this nutrition is administered during surgery, which has been found to be most effective in experimental studies. We are not aware of previous, current or proposed studies with similar innovative approaches. To ensure patient safety this study is conducted according to the rules of Good Clinical Practice and a Data Safety Monitoring Board is installed.

Improvement of postoperative care and reduction of POI and AL is a complex challenge that requires a multidisciplinary approach. This clinical study will be performed based on the hypothesis that early modulation of the immune response by enriched enteral nutrition will reduce POI and also affect AL. A reduction of postoperative morbidity by this nutritional intervention may contribute to an enhanced efficiency, resulting in measurable improvements of productivity, cost and quality.

In conclusion, this study will investigate whether nutrition can be used to reduce POI and AL following colorectal surgery. Nutrition will be administered as a means to stimulate the autonomic nervous system and to modulate the immune response directly. Furthermore, the timing is essential for the magnitude of effect. Reduction of POI and AL following colorectal surgery would not only optimize surgical care for this large patient group and thereby reduce healthcare costs, but may also open new therapeutic opportunities and changes the way of thinking about the usage of enteral nutrition in a clinical setting.

## Trial status

Open for inclusion since August 2014.

## Abbreviations

AL: anastomotic leakage
CCK: cholecystokinin
DSMB: Data Safety Monitoring Board
ELISA: enzyme-linked immunosorbent assay
en%: energy percent
ERAS: Enhanced Recovery After Surgery
EQ-5D-5L: EuroQol-5D-5L
ICER: incremental cost-effectiveness ratio
ICUR: incremental cost-utility ratio
PCR: polymerase chain reaction
POI: postoperative ileus
QALYs: quality-adjusted life years
TNF-α: tumor necrosis factor alpha
